# Osthole/borneol thermosensitive gel via intranasal administration enhances intracerebral bioavailability to improve cognitive impairment in APP/PS1 transgenic mice

**DOI:** 10.3389/fphar.2023.1224856

**Published:** 2023-07-13

**Authors:** Fanchang Wu, Mingjun Huang, Xue Zuo, Ruiye Xie, Jinman Liu, Junyu Ke, Weirong Li, Qi Wang, Yong Liang

**Affiliations:** ^1^ Science and Technology Innovation Center, Guangzhou University of Chinese Medicine, Guangzhou, China; ^2^ Institute of Clinical Pharmacology, Guangzhou University of Chinese Medicine, Guangzhou, China; ^3^ Affiliated Jiangmen TCM Hospital of Jinan University, Jiangmen, China; ^4^ School of Basic Medical Sciences, Guangzhou University of Chinese Medicine, Guangzhou, China

**Keywords:** osthole, borneol, intranasal administration, bioavailability, APP/PS1 mice, thermosensitive gel

## Abstract

Alzheimer’s disease (AD) poses a significant threat to the global elderly population. Traditional Chinese medicine (TCM) has been widely utilized in the treatment of AD. Osthole, a bioactive ingredient classified as an “emperor” in many TCM formulas, has been demonstrated to effectively alleviate AD symptoms. However, its low bioavailability in the brain has limited its clinical application. This study aimed to increase the intracerebral bioavailability of osthole by using borneol as a “courier,” based on the classical “Emperor–Minister–Assistant–Courier” model, and to investigate the enhanced pharmacological performance of osthole on AD. Results indicated that a suitable *in situ* thermosensitive gel matrix for intranasal administration mixed with osthole and borneol consists of P407 at 20%, P188 at 7%, and PEG300 at 6%. The concentration of osthole in the cerebrospinal fluid increased almost tenfold after intranasal administration of osthole/borneol compared to oral administration. Mechanisms showed that borneol as a “courier” opened up intercellular space and loosened the tight junctions of the nasal mucosa by suppressing ZO-1 and occludin expression, thereby expediting the nose-to-brain route and guiding osthole as “emperor” to its target in the brain. Osthole assisted by borneol demonstrated significantly improved efficiency in suppressing cleaved caspase-3 expression, increasing the Bcl-2/Bax ratio, improving T-SOD and catalase expression, reducing malondialdehyde levels, inhibiting neuron apoptosis, and decreasing Aβ levels by inhibiting BACE1 expression to alleviate cognitive impairment in APP/PS1 mice compared to osthole alone. Overall, our study demonstrated that the intracerebral bioavailability of osthole profoundly improved with intranasal administration of osthole/borneol and provided a wider application of TCM for AD treatment with higher intracerebral bioavailability.

## 1 Introduction

As the global population continues to age, Alzheimer’s disease (AD) has emerged as a major health threat to the elderly due to its high morbidity and mortality. AD is the most common neurocognitive disorder, affecting 46.8 million people, and is projected to impact 131.5 million by 2050 (Khoury et al, 2017). The clinical symptoms of AD are characterized by progressive cognitive decline and multiple pathological features, including neural inflammation, mitochondrial dysfunction, autophagy dysfunction, apoptosis, oxidative stress, tau protein hyperphosphorylation, and β-amyloid (Aβ) plaque deposition (Agrawal et al, 2018; Khan et al, 2020). Despite advances in biomedical research, only five drugs have been approved by the US Food and Drug Administration (FDA) to clinically treat AD. Four of these—tacrine, rivastigmine, galantamine, and donepezil—are acetylcholinesterase inhibitors. Tacrine, the first drug approved for mild to moderate AD, was discontinued in the United States in 2013 due to its hepatotoxicity and uncertain efficacy (Galisteo et al, 2000; Pan et al, 2011). Currently, most drugs used to treat AD provide only temporary relief and are unable to halt or reverse the progression of the disease (Se Thoe et al, 2021). Given the complex pathogenesis of AD, there remains a pressing need for effective treatments.

Traditional Chinese medicine (TCM) herbal formulas have been widely used for preventing and treating neurodegenerative diseases like AD and Parkinson’s disease (Pei et al, 2020). Due to classical compatibility theory and their synergistic properties, TCM formulas can reduce adverse effects and enhance therapeutic efficacy in treating diseases like AD (Luan et al, 2020). One of the most classical rules in TCM formulas is the “Emperor–Minister–Assistant–Courier” theory, known as *jun–chen–zuo–shi* in Chinese. The bioactive constituents in TCM formulas act as “emperors,” whose primary therapeutic effect is to treat the cause of disease. Other ingredients act as “couriers,” which assist the “emperor” to deliver drugs to pathogenic organs, particularly those in upper parts of the body such as the brain (Zheng et al, 2018). For example, *Rehmannia glutinosa Libosch* acts as an “emperor” in the Liuwei-Dihuang decoction to tonify the kidney and nourish *qi*, thus exhibiting benefits in ameliorating AD pathologies and behavioral deficits (Cheng et al, 2020). Another classic formula, Kai-Xin-San, also displays profound therapeutic effects on dementia (Jiao et al, 2022), with *Panax ginseng* C. A. Mey as “emperor” and *Acorus tatarinowii* Schott as “courier”.

Borneol, also known as *bing-pian* or *long-nao* in Chinese, has been used as a TEC messenger or courier for approximately 1,000 years (Yang et al, 2009; Mei et al, 2023). This bicyclic monoterpene, classified as camphene, has a distinct fragrance and is highly lipid-soluble. In TCM, borneol is primarily used as a “courier” in treating various central nervous system (CNS) disorders, including dementia, stroke, cerebral ischemia, and cerebritis. The Bushen-Yizhi formula (BSYZ), comprising osthole (OST) as “emperor” and borneol as “courier,” exerts an effect to enhance learning and memory capabilities (Hou et al, 2015; Zhang et al, 2017; Cai et al, 2018; Chu et al, 2020). Recent findings indicate that the bioavailability of OST in rat plasma significantly increased after oral administration of BSYZ extract compared to pure osthole at the same dose (Liu et al, 2022). These results further demonstrate the rationality of the Emperor–Minister–Assistant–Courier theory in TCM and promote the prevention and treatment of AD.

The relatively poor bioavailability of active constituents in the brain has severely limited the further application of TCM formulas for AD. The first difficulty that impairs drug bioavailability is the well-known hepatic and intestinal first-pass effect, where drugs are metabolized and pumped out by hepatic and intestinal CYP450 enzymes and efflux proteins such as P-glycoprotein and various multidrug resistance proteins (Gao et al, 2020). Additionally, the blood–brain barrier (BBB) restricts drug bioavailability in the brain, presenting another critical deficiency for AD treatment (Rajput et al, 2022). The combined obstacles of the first-pass effect and BBB significantly reduce drug concentration. Even when guided by borneol, the improved bioavailability in the brain remains limited (Gao et al, 2010; He et al, 2011). Therefore, enhancing intracerebral bioavailability is essential to increasing therapeutic effects on brain diseases.

In this study, osthole (OST) was used as a demonstrative example, assisted by borneol (BO) via intranasal administration to significantly enhance the bioavailability of OST in the brain (Erdő et al, 2018). Based on the Emperor–Minister–Assistant–Courier theory, we constructed an *in situ* thermosensitive gel mixed with OST as the “emperor” and BO as the “courier” for intranasal administration. The experimental conditions of gelation and drug release were optimized. The pharmacokinetics of OST in plasma and cerebrospinal fluid (CSF) of rats via intranasal and oral treatment were comparatively evaluated. The possible mechanisms of BO in the OST/BO gel that guided OST to the brain were further investigated. Finally, APP/PS1 transgenic mice were used to verify the enhanced pharmacological effects of the OST/BO gel on AD. The behavioral test of APP/PS1 mice and related biological processes were extensively evaluated to demonstrate the advantageous features of the OST/BO gel in improving cognitive impairment. It is expected that the results of this study will reveal new insights into enhancing intracerebral bioavailability, benefiting TCM for AD treatment. Moreover, this work should promote an understanding of the Emperor–Minister–Assistant–Courier theory and provide modern scientific confirmation of TCM compatibility.

## 2 Materials and methods

### 2.1 General information on materials

All the reagents in this study were at least analytical grade except where stated. Ultrapure water was prepared using the RODI-220A system with 18 MΩ cm sensitivity (Guangzhou Ewell Bio-technology Co., China). Formic acid, methanol, HPLC-grade acetonitrile, phosphate-buffered saline (PBS), and (−) borneol (99.9%) were purchased from Aladdin Biochemical Technology Co., Ltd. (Shanghai, China). Ethyl acetate was bought from Sigma Aldrich (Shanghai, China). Poloxamer 407 (P407) and 188 (P188) were purchased from Bidepharm (Guangzhou, China). Osthole (99.9%) and PEG300 were bought from Macklin (Shanghai, China). Imperatorin (IMP, 99.9%) was purchased from Tauto Biotech (Shanghai, China). Glyceraldehyde-3-phosphate dehydrogenase (GAPDH, #GB11002) was obtained from Servicebio, China. Goat anti-mouse IgG (H + L) Fluor488-conjugated (#S0017) and goat anti-rabbit IgG (H + L) fluor594-conjugated (#S0006) were bought from Affbiotech (Shanghai, China). DAPI stain solution was bought from YEASEN (Shanghai, China). The malondialdehyde (MDA) (#A003-1-2), catalase (CAT) (#A007-1-1), and total superoxide dismutase (T-SOD) assay kits (#A001-1-2) were purchased from Nanjing-Jiancheng Bio (Nanjing, China). Rabbit ZO-1 antibody (#TA5145), occludin antibody (#T55997) for immunofluorescence, occludin antibody (TD7504) for Western blot, rabbit anti-BACE1 antibody (#T56880), anti-B-cell lymphoma-2 antibody (Bcl-2, #T40056), anti-Bcl-2-associated X antibody (Bax, T40051), anti-cleaved caspase 3 (TA7022), goat anti-mouse IgG HRP (#M21001), and goat anti-rabbit IgG-HRP (#M21002) antibodies were all bought from Abmart (Shanghai, China).

### 2.2 Animals

For the pharmacokinetic study of OST in rat plasma and cerebrospinal fluid, male Sprague–Dawley rats (SD rats, 300 ± 20 g) were purchased from Guangzhou University of Chinese Medicine Animal Laboratory Center. In order to further address the enhanced pharmacological effects of OST/BO gel with nasal administration, 4-month-old male APP/PS1 transgenic mice (Varga-Medveczky et al, 2021) were purchased from Beijing Hfk Bioscience Co., Ltd. (Beijing, China) (certification number SCXK 2014-0004). A control of 4-month-old male C57BL/6J mice were also bought from Guangzhou University of Chinese Medicine Animal Laboratory Center. The animals were housed in a room with a controlled temperature of 24°C ± 2°C, a 12/12 h light/dark cycle, and a relative humidity of 55%–60%. They were provided with *ad libitum* access to a standard rodent diet and drinking water.

All animal procedures were in accordance with the Regulations on the Administration of Experimental Animals issued by the Ministry of Science and Technology of the People’s Republic of China. Animal welfare and experimental procedures were also strictly approved with the guide for the care and use of laboratory animals and related ethical regulations of the Experimental Animal Center of Guangzhou University of Chinese Medicine (ethical number: 20220329002).

### 2.3 Preparation of OST/BO thermosensitive gel

P407, P188, and additives are commonly used to prepare suitable temperature-sensitive gels; we mainly followed our previous procedure with some modifications (Chen et al, 2021). Briefly, with the help of the Box–Behnken design, 20%–24% P407 and 3%–10% P188 were verified and then added with an applicable amount of deionized water to form the gel mixture. This mixture was stirred at 4°C until the dispersion was uniform; a ratio of 0%–12.5% PEG300 was then added and stirred continually. The gel mixture was stored at 4°C for more than 24 h to fully dissolve the components. Afterward, 1 mL gel was transferred to a 10-mL vial and incubated in a water bath whose temperature rose slowly from 20°C to 35°C at a rate of 1°C/min. The temperature at which the liquid no longer flowed was recorded as the gelation temperature, and that time was deemed the gelation time. The optimal formula was chosen based on the data analyzed using Design-Expert software (Version 10.0.7). Finally, OST and BO (dissolved in 2% DMSO) were decanted into the optimal gel matrix to assemble the OST and OST/BO thermosensitive gels.

### 2.4 OST release in thermosensitive gel

Before subsequent intranasal administration, the release of OST in OST/BO and OST thermosensitive gel was simulated *in vitro*. It was performed on a membrane-less dissolution model. First, 5 mL OST/BO or OST gel was deposited in a centrifuge tube at 30°C. The tube was then incubated in 40 mL PBS in a shaker water bath, set at 150 rpm and 33°C for 3 h. Samples were withdrawn at 30, 60, 120, 180, 240, and 300 min, wherein an equal volume of PBS buffer was supplemented to maintain the sink conditions. The concentration of OST in samples was measured by LC-MS/MS using IMP as an internal standard, following Liu et al. (2022).

### 2.5 Comparative pharmacokinetic study of OST in rat CSF and plasma under intranasal and oral administration

For intranasal administration, OST and OST/BO gel with two doses (1.25 and 2.5 mg/kg) were exploited. The solubility of OST or BO in gel was determined to be 12.5 mg/mL. A volume of 500 μL of OST in corn oil (1.25 mg/mL) was administered via oral gavage, resulting in a total dose of 0.625 mg. For intranasal administration, a volume of 50 μL of OST in gel (12.5 mg/mL) was used in the right nasal nostril, also resulting in a total dose of 0.625 mg. Assuming the weight of the rat to be 250 g, the calculated dose was 2.5 mg/kg. The SD rats were fasted for 12 h and randomly divided into four groups with six rats in each (*n* = 6)—Group I: OST (1.25 mg/kg in gel), Group II: OST/BO (1.25 mg/kg for each in gel), Group III: OST (2.5 mg/kg in gel), and Group IV: OST/BO (2.5 mg/kg for each in gel). For oral administration, OST and OST/BO were dissolved in corn oil, with two doses tested (1.25 and 2.5 mg/kg OST). SD rats were similarly subjected to a 12-h fast and were randomly divided into four groups of six rats per group (*n* = 6)—Group Ⅴ: OST (1.25 mg/kg, in corn oil), Group Ⅵ: OST/BO (1.25 mg/kg for each, in corn oil), Group Ⅶ: OST (2.5 mg/kg, in corn oil), and Group Ⅷ: OST/BO (2.5 mg/kg for each, in corn oil).

For CSF sampling, the operational process improved on the method of Nirogi et al. (2009) by connecting two blood collection needles. The front end of the needle could not move after insertion into the brain of rats, while the back end of the needle could transfer the extracted CSF at different times. In this way, CSF had a high probability of avoiding contamination by blood infiltration of peripheral tissues. Some 40 μL of CSF was sampled from a depressible surface between the occipital protuberances and the spine of the atlas at 30, 60, 120, 180, and 240 min after oral and intranasal administration (*n* = 6). In terms of plasma, 300 μL blood was sampled from the infraorbital vein into a heparinized tube at 5, 15, 30, 60, 120, 180, 240, 300, and 360 min after oral administration (*n* = 6). All CSF and blood samples were immediately centrifuged at 4°C, 3,500 rpm, and 10 min, and then the supernatant was discarded and stored at −80°C until analysis. The concentration of OST in CSF and plasma was measured by LC-MS/MS using IMP as the internal standard (Liu et al, 2022).

### 2.6 Behavioral test

#### 2.6.1 Animal groups

For the further pharmacological study of OST/BO gel with intranasal administration, C57BL/6J 7-month-old mice were set as the WT group, and 7-month-old APP/PS1 transgenic mice were randomly divided into four groups with two doses of OST treatment—Group Ⅰ: APP/PS1 (gel matrix), Group Ⅱ: OST (2.5 mg/kg in gel), Group Ⅲ: OST/BO (1.25 mg/kg OST and 2.5 mg/kg BO, in gel), and Group Ⅳ: OST/BO (2.5 mg/kg for each, in gel). The animals were intranasally administered with OST and OST/BO gel (*n* = 6). After 6 weeks of continuous intranasal treatment, the mice’s behavioral tests were conducted, including open field, novel objective recognition, Y-maze, and Morris water maze tests.

#### 2.6.2 Open field test (OFT)

The OFT was conducted to detect the spontaneous activity of APP/PS1 mice. They were placed in the center of a square arena (47 cm × 47 cm × 40 cm), and their movements were monitored using an infrared light photo beam system for a duration of 5 min. The movement was then time analyzed using XinRuan software (Shanghai XinRuan Information Technology Co., Ltd.).

#### 2.6.3 Novel object recognition test (NORT)

On the first day, the tested mice (*n* = 6) were habituated to the testing area for 10 min. On the second day, they were placed in an opaque box with two objects (A and B) to explore for 5 min before being returned to their cage. After an hour, the B object was replaced with C in the same position, and the mice were returned to the box to explore for another 5 min. Data were collected using XinRuan software, and the recognition index was analyzed by dividing the time spent exploring the novel object by the total exploration time of both objects.

#### 2.6.4 Y-maze test

In brief, the mice were placed at the end of one arm and allowed to freely navigate through the maze for 5 min. For an arm entry to be counted, all four paws had to be within the arm. To achieve a perfect alternation, the mice had to enter three different arms in overlapping sets of three successive entries. The total number and sequence of arm entries were recorded, and data were collected using XinRuan software. The percentage of spontaneous alternation behaviors (SAB) (%) was calculated using the formula [successive triplet sets (consecutive entries into three different arms)/total number of arm entries-2] × 100% (Ali et al, 2015). The maze was cleaned with 70% ethanol after each test to remove residual odors.

#### 2.6.5 Morris water maze (MWM) test

The MWM test was conducted in a dimly lit room using a circular tank with a diameter of 140 cm and height of 50 cm. A submerged escape platform measuring 10 × 10 cm was placed 1.5 cm below the milky water surface in one of the quadrants. Spatial cues of different geometry were placed along the sides of the pool to assist the mice in recognizing the platform position. The mice were individually handled for 1 day before undergoing a 5-day acquisition training with four trials per day. A trial was considered complete once the mice found the platform or after 60 s had elapsed. If they were unable to find the submerged platform during a trial, they were guided to it. The latency and path to the platform were tracked and recorded, and swim speed was measured to analyze the involvement of motor function as a confounding factor. On Day 6, a single probe test was performed 24 h after the last trial of the acquisition phase to measure the integrity and strength of spatial memory. XinRuan software was used to collect the results of the probe trial, including the time spent in the given quadrant and the average proximity to the escape annulus.

### 2.7 HE staining of nasal mucosa

After transcardial perfusion with 0.9% saline followed by 4% paraformaldehyde, the nasal tissues of SD rats and APP/PS1 mice were collected and post fixed with 4% paraformaldehyde overnight. These tissues underwent 10% EDTA decalcification for 2 weeks, then were dehydrated, paraffin-embedded, and sectioned. The tissue sections were dewaxed with xylene and rehydrated with ethanol and were then stained with HE dye in proper order. The images were obtained using a TE 2000U microscope (Nikon, Japan).

### 2.8 Immunofluorescence assay

The paraffin sections of SD rats and APP/PS1 mice were dewaxed with xylene and rehydrated with gradient ethanol. Tissue sections were heated with a sodium citrate antigen recovery buffer for 25 min. They were blocked with bovine serum albumin (BSA) for 30 min and then incubated with occludin (dilution 1:100) and ZO-1 (dilution 1:100) antibodies in a humidified box at 4°C overnight. The sections were incubated the next day with fluorescein-labeled secondary antibody out of light for 1 h at 37°C. The nuclei were stained with DAPI stain solution for 3 min. The images were visualized using a TE 2000U (Nikon, Japan); red fluorescence represented ZO1, green represented occludin, and blue fluorescence represented cell nuclei.

### 2.9 Western blot (WB) assay

In WB experiments, the nasal mucosa of SD rats and the hippocampus of APP/PS1 transgenic mice were extracted with cold lysis buffer (Solarbio, China) containing 1 mmol/L phenylmethyl sulfonyl fluoride. The total protein concentration was measured using the bicinchoninic acid (BCA) protein assay kit (YEASEN, China). The proteins were separated by SDS-PAGE (8%–12%) and transferred onto polyvinylidene difluoride membranes. After blocking non-specific binding sites with 5% dried skim milk for 2 h, the membranes were incubated with primary antibodies overnight at 4°C. Specific antibody binding was detected by incubating with horseradish peroxidase-conjugated secondary antibody for 2 h at room temperature. Protein expression was detected using the enhanced chemiluminescence method with a Bio-Spectrum Gel Imaging System (UVP, United States). The relative expression of target proteins was normalized to that of GAPDH. ZO-1 (dilution 1:1000), occludin (dilution 1:1000), Bax (dilution 1:1000), Bcl-2 (dilution 1:1000), BACE1 (dilution 1:1000), GAPDH (dilution 1:1000), cleaved caspase 3 (dilution 1:1000), goat anti-mouse IgG HRP (dilution 1:5000), and goat anti-rabbit IgG-HRP (dilution 1:5000) were used.

### 2.10 Nissl staining

The brain tissues of the mice in each group were carefully removed and rapidly fixed in 4% paraformaldehyde (dissolved in 100 mM PBS) for 24 h. They were then dehydrated in ethanol and embedded in paraffin blocks in sections 3 µm thick using a paraffin tissue slicer (Leica Biosystems, Germany). The brain tissues were subsequently dewaxed with xylene and rehydrated with ethanol, followed by staining with Nith’s solution as per instructions (#C0117, Beyotime, China). The neurons and Nissl bodies in the hippocampus were observed using a biological microscope, and the images were collected and analyzed using ImageJ software.

### 2.11 Thioflavin S (Th-S) staining

To detect amyloid plaques in brain tissue sections, the slices were first dewaxed with xylene and rehydrated using a gradient of ethanol. They were then incubated with a solution of 0.3% thioflavin-S in 50% distilled ethyl alcohol in the dark for 8 min at room temperature. Following differentiation in 70% ethanol and washing with distilled water, the slices were fixed with 50% glycerin in PBS. Finally, they were visualized using a TE 2000U microscope (Nikon, Japan).

### 2.12 Measurement of MDA, T-SOD, and CAT in hippocampus

The brain tissues were carefully removed and placed on ice trays for dissection into the hippocampus, then rapidly frozen and stored at −80°C until use. The selected hippocampus tissues were immediately placed in ice-cold PBS to wash away blood and remove fibers and fat. After filtering by 300-mesh nylon mesh, the cell suspension was collected and washed with ice-cold PBS. After centrifuging at 500 rpm for 5 min and discarding the supernatant, the sediment was washed twice with PBS. The hippocampal tissues were homogenized in cold normal saline and centrifuged at 12,000 rpm for 10 min at 4°C. Then, a BCA protein assay kit (YEASEN, China) was used to detect the protein concentration. The concentrations of soluble/insoluble MDA, T-SOD, and CAT were measured according to the manufacturer’s instructions.

### 2.13 Statistical analysis

Pharmacokinetic parameters were determined using Drug and Statistics software (version 3.0, Shanghai Bojia Pharmaceutical Technology Co., Ltd., Shanghai, China). Statistical analyses were performed using GraphPad Prism software (version 8.0, United States). One-way ANOVA and Tukey’s *post hoc* test were used to examine the mean differences across multiple groups. The results were presented as mean ± standard deviation (SD). *p* < 0.05 was considered statistically significant, and *p* < 0.01 and 0.001 were very statistically significant.

## 3 Results

### 3.1 The most suitable thermosensitive gel formula and properties of OST release

**FIGURE 1:**
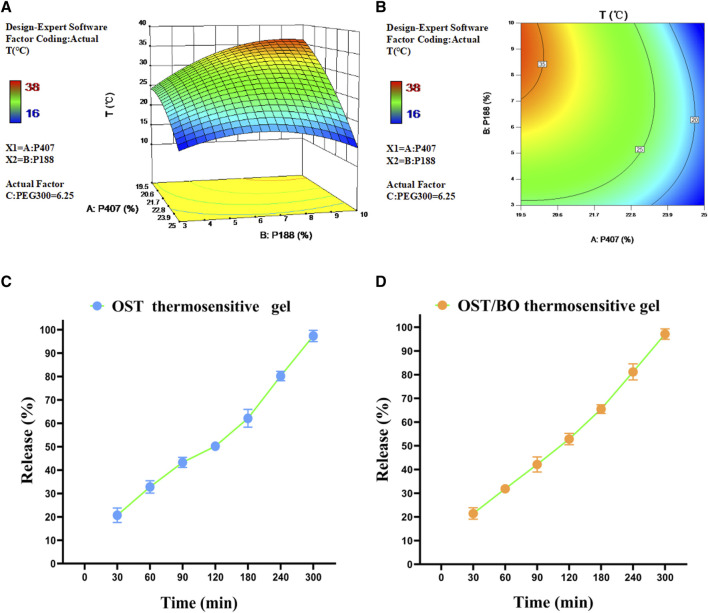
**(A)** Three-dimensional and **(B)** two-dimensional response surface of P407, P188, and PEG300 as well as gelation temperature in thermosensitive gel with PEG300 = 6.25% using Design-Expert 10.0.7 software. Release behavior of OST in **(C)** OST and **(D)** OST/BO thermosensitive gel.

**FIGURE 2:**
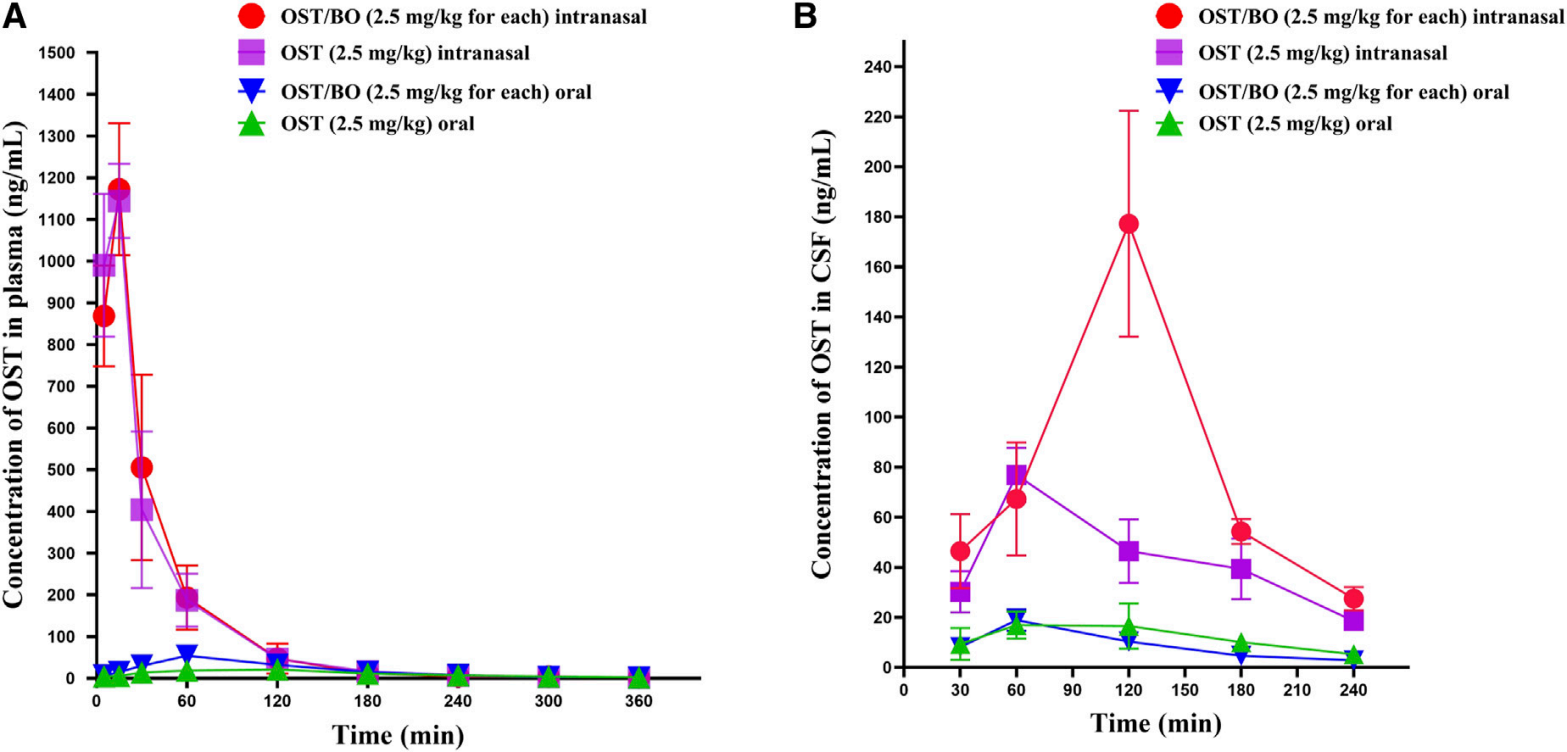
Curve of average concentration for OST (ng/mL) vs. sampling time (min) in rat plasma and CSF determined by LC-MS/MS. **(A)** Content of OST in rat plasma after oral and intranasal administration (*n* = 6). **(B)** Content of OST in rat CSF after oral and intranasal administration (*n* = 6).

The three factors of P407 (20%–24%), P188 (3%–10%), and PEG300 (0%–12.5%), as well as gelling temperature (T) as a dependent variable, were optimized (Supplementary Table S1). Design-Expert 10.0.7 software was used to perform a non-linear fitting analysis on the data. The 3D effect response surface (Figure 1A) and 2D contour response surface (Figure 1B) were drawn with the quadratic polynomial equation as a mathematical model. The quadratic polynomial fitting equation of gelling temperature was obtained as follows: Y = 14.70776X1 + 12.78028X2 + 0.58845X3 − 0.33522X1X2 − 0.067360X1X3 − 0.060438X2X3 − 0.33501X1^2^ − 0.32119X2^2^ + 0.083191X3^2^ − 148.55684 (*p* < 0.05). Y denotes gelation temperature, X1 is P407, X2 is P188, and X3 is PEG300. From the light-yellow area of three dimensional contour (Figure 1A), the ideal gelation temperature was 32–34°C. P407 = 20%, P188 = 7%, and PEG300 = 6% in gel matrix were selected due to the physiological temperature of 33°C in the nasal cavity.

After optimization, the maximum solubility was 12.5 mg/mL for both OST and BO in gel. The dissolution and release of OST in OST and OST/BO gel were investigated by the membrane-less dissolution method *in vitro* (Figures 1C, D). The OST cumulative dissolution rate in OST gel within 300 min was greater than 95%, and the released amount positively correlated with time (Figure 1C). As for OST/BO gel, the cumulative dissolution rate of OST was greater than 95% within 300 min and also correlated with time (Figure 1D). Behind these results, OST was almost completely released in thermosensitive gel capable of intranasal administration in animals.

### 3.2 Pharmacokinetics study of OST in rat plasma and CSF via oral and intranasal administration

First, the pharmacokinetics of OST in rat plasma was evaluated under oral and intranasal administration (Figure 2A). With OST (2.5 mg/kg in gel) intranasally administered, the AUC_0-t_ value of OST in rat plasma was (43,653 ± 6,539)—significantly higher than that of oral administration (2.5 mg/kg in corn oil) (3,948 ± 601) (*p* < 0.01). As for OST/BO gel intranasally treated (2.5 mg/kg for each), the AUC_0-t_ value of OST in plasma was (45,405 ± 1711) compared with that of oral administration (2.5 mg/kg for each, in corn oil) (6,907 ± 985) (*p* < 0.01) (Table 1). The same phenomenon was also found using a lower dose of OST (1.25 mg/kg) and OST/BO (1.25 mg/kg for each) via oral and intranasal administration (Supplementary Figure S1), which were inferior to the high dose (2.5 mg/kg).

**TABLE 1:** Pharmacokinetic study of OST in rat plasma under oral and intranasal administration (*n* = 6).

Group	OST (2.5 mg/kg) oral	OST (2.5 mg/kg) intranasal	OST/BO (2.5 mg/kg for each) oral	OST/BO (2.5 mg/kg for each) intranasal
AUC_0-t_ (ng/L*min)	3,948 ± 601	43,653 ± 6539**	6,907 ± 985	45,405 ± 1711^##^
AUC_0-∞_ (ng/L*min)	4,170 ± 610	43,771 ± 6461**	7,009 ± 1,050	45,555 ± 1602^##^
MRT_0-t_ (min)	135.4 ± 15.6	39.0 ± 1.9	108.5 ± 7.7	38.6 ± 7.1
MRT_0-∞_ (min)	152.7 ± 23.9	40.27 ± 1.8	113.3 ± 11.4	40.0 ± 6.9
T_1/2_ (min)	64.8 ± 12.7	51.0 ± 13.5	45.4 ± 19.3	59.9 ± 13.5
Vz/F (L/kg)	57,199 ± 16,416	4,357 ± 1,607	23,179 ± 9,269	4,762 ± 1,212
CLz/F (L/min/kg)	607.5 ± 82.4	57.9 ± 8.2**	361.7 ± 49.9	54.9 ± 1.9^##^
C_max_ (ng/L)	22.5 ± 0.5	1,144.5 ± 89.0***	54.2 ± 10.6	1,172.6 ± 158.3^###^

*OST (2.5 mg/kg in gel) intranasal administration group compared with OST (2.5 mg/kg in corn oil) oral administration group; ^#^OST/BO (2.5 mg/kg in gel) intranasal administration group compared with OST/BO (2.5 mg/kg in corn oil) oral administration group, *p < 0.05, **p < 0.01, ***p < 0.001.

Next, the pharmacokinetics of OST in CSF was studied under oral and intranasal administration (Figure 2B). The AUC_0-t_ values of OST in CSF was (10,083 ± 1,158) via intranasal administration (OST, 2.5 mg/kg in gel), which was significantly improved over the oral route (2,811 ± 703) (*p* < 0.01) (OST, 2.5 mg/kg in corn oil). When OST/BO gel was used (2.5 mg/kg for each), the AUC_0-t_ values of OST in CSF was (19,148 ± 3,363) via intranasal administration, almost ten times higher than oral administration (2.5 mg/kg for each, in corn oil) as (2090 ± 278) (*p* < 0.01) (Table 2). For lower doses of OST (1.25 mg/kg) and OST/BO gel (1.25 mg/kg for each) via either oral or intranasal treatment, the same tendency of OST in CSF was found (Supplementary Figure S1). From the results (Figure 2B), the concentration of OST in CSF was highly increased with OST/BO gel intranasally treated compared to OST gel alone (*p* < 0.01). In addition, OST in rat plasma almost exhibited no difference under either OST or OST/BO gel intranasal treatment; the function of BO in OST nasal delivery is explored in the following section.

**TABLE 2:** Pharmacokinetic study of OST in rat CSF under oral and intranasal administration (*n* = 6).

Group	OST (2.5 mg/kg) oral	OST (2.5 mg/kg) intranasal	OST/BO (2.5 mg/kg for each) oral	OST/BO (2.5 mg/kg for each) intranasal
AUC_0-t_ (ng/L*min)	2,811 ± 703	10,083 ± 1158***	2090 ± 278	19,148 ± 3362^###&&^
AUC_0-∞_ (ng/L*min)	3,547 ± 1,135	12,448 ± 1685***	2,377 ± 295	28,167 ± 19,055^###&&^
MRT_0-t_ (min)	112.1 ± 14.4	111.7 ± 6.2	98.2 ± 9.2	119.9 ± 5.1
MRT_0-∞_ (min)	150.1 ± 33.7	159.7 ± 22.1	128.3 ± 13.5	186.0 ± 110.4
T_1/2_ (min)	81.8 ± 25.2	86.8 ± 15.2	69.6 ± 15.2	141.2 ± 227.0
Vz/F (L/kg)	86,867 ± 29,137	25,231 ± 3,354	107,306 ± 29,386	12,801 ± 10,031
CLz/F (L/min/kg)	755.0 ± 193.6	204.2 ± 29.6***	1,066.3 ± 141.3	109.5 ± 38.0^###&^
C_max_ (ng/L)	21.8 ± 6.1	76.9 ± 10.8***	18.9 ± 4.3	177.2 ± 45.1^###&&^

*OST (2.5 mg/kg in gel) intranasal administration group compared with OST (2.5 mg/kg in corn oil) oral administration group; ^#^OST/BO (2.5 mg/kg for each) intranasal administration group compared with OST/BO (2.5 mg/kg for each) oral administration group; ^&^OST/BO (2.5 mg/kg for each) oral intranasal administration group compared with OST (2.5 mg/kg) intranasal administration group, *p < 0.05, **p < 0.01, ***p < 0.001, ^#^ *p* < 0.05, ^##^ *p* < 0.01, ^###^ *p* < 0.001, ^&^ *p* < 0.05, ^&&^ *p* < 0.01, ^&&&^ *p* < 0.001 (*n* = 6).

### 3.3 Effects of borneol on nasal mucosa

**FIGURE 3:**
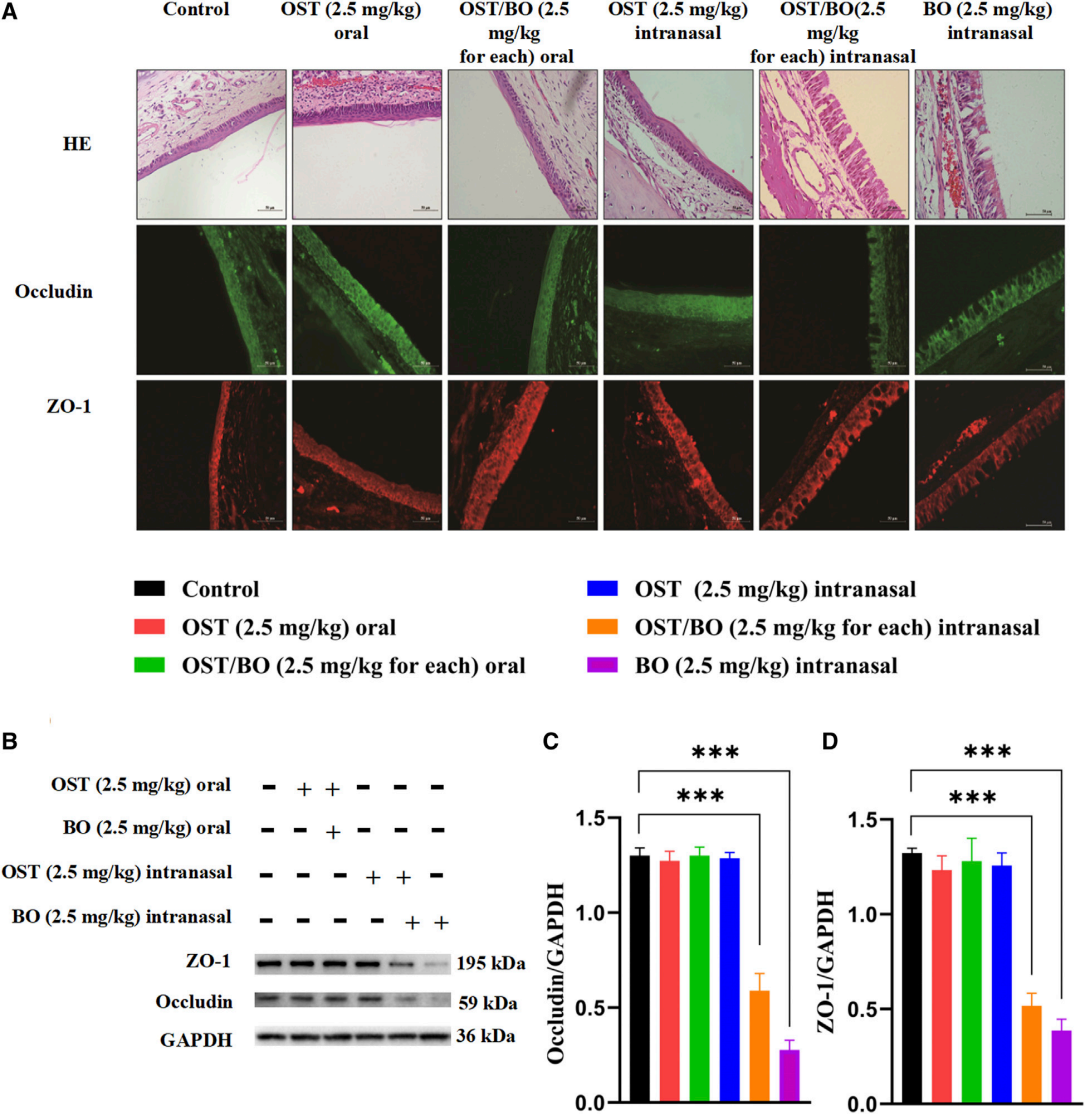
**(A)** Pathological images of nasal mucosa stained with H&E and immunohistochemistry images of ZO-1 and occludin in nasal mucosa. **(B)** WB bands of ZO-1 and occludin in nasal mucosa. **(C,D)** Statistical analysis of ZO-1 and occludin in nasal mucosa from WB. Statistical significance denoted as *p < 0.05, **p < 0.01, ***p < 0.001 (*n* = 6).

In terms of HE staining, nasal mucosa morphology remained intact in all SD rat groups (Figure 3A). Compared with the controls, the intercellular space of nasal mucosa was obviously divided after OST/BO (2.5 mg/kg, in gel) and BO (2.5 mg/kg, in gel) intranasal administration; however, there was not so much morphological change in oral administration of OST and OST/BO as well as of OST alone with the intranasal route. The tight junctions in the nasal mucosa were opened up after OST and OST/BO intranasal administration, according to immunofluorescence assay (Figure 3A). The results of WB further revealed that the expressions of ZO-1 and occludin in OST/BO and BO intranasal administration groups were visibly lower than for oral groups (*p* < 0.05) (Figures 3–D).

### 3.4 Behavioral test of APP/PS1 mice via intranasal administration of OST/BO thermosensitive gel

**FIGURE 4:**
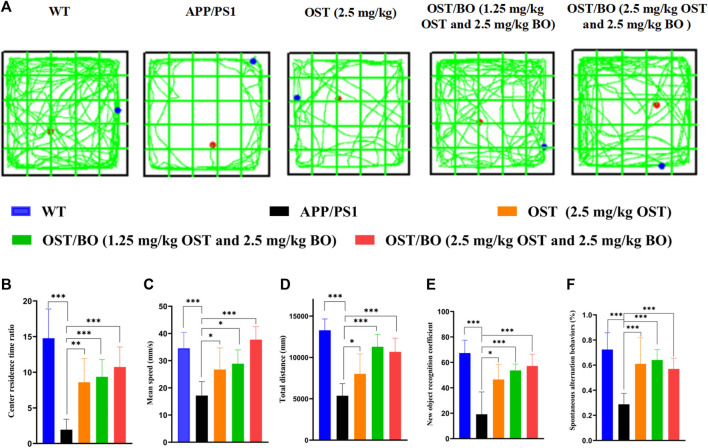
**(A)** Activity trajectory of each group in the OFT, where the red points indicate the starting points and the blue points indicate the ending points. **(B)** Statistical analysis of **(B)** center residence time ratio, **(C)** mean speed, **(D)** total distance, **(E)** new object recognition coefficient, and **(F)** percentage of SAB. Statistical significance denoted as *p < 0.05, **p < 0.01, ***p < 0.001, (*n* = 6).

**FIGURE 5:**
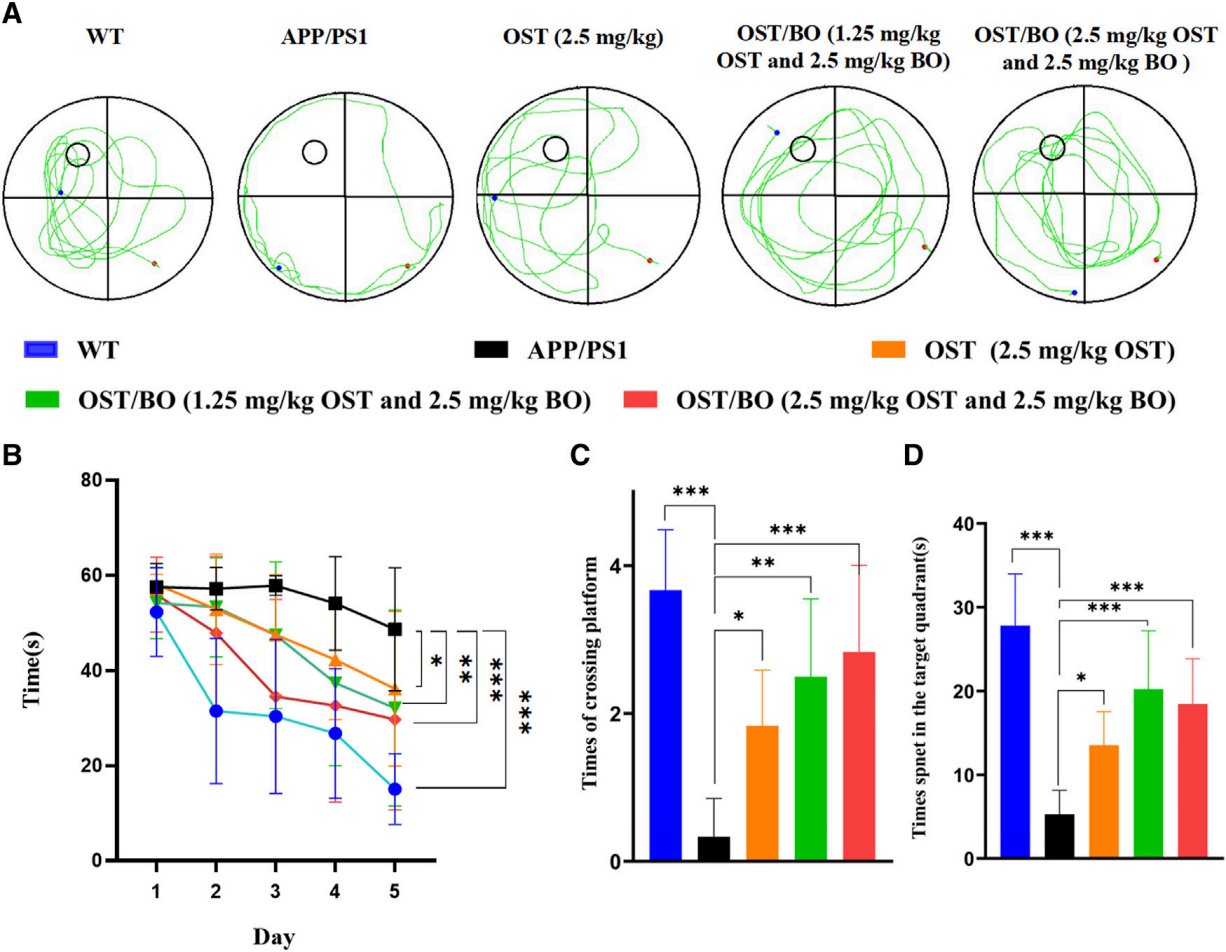
**(A)** Tracings of animal paths during the positioning navigation test on Day 6. Statistical analysis of **(B)** escape latencies in water maze during the positioning navigation test, **(C)** number of times of crossing the platform location during the probe trial, and **(D)** time spent in the target quadrant during the probe trial. Statistical significance denoted as *p < 0.05, **p < 0.01, ***p < 0.001, (*n* = 3).

The behavioral test was first evaluated from OFT experiments. Compared with the WT group, the APP/PS1 transgenic mice were troubled by severe motor dysfunction. Their locomotor activity and exploratory behavior significantly improved after intranasal administration of OST (2.5 mg/kg, in gel), lower OST/BO (1.25 mg/kg OST, in gel), and OST/BO (2.5 mg/kg OST, in gel) (Figure 4A). The total distance was increased by 49.51% (*p* < 0.05) with OST gel intranasal administration, 110.31% (*p* < 0.001) with lower OST/BO, and 98.82% (*p* < 0.001) with OST/BO intranasal administration (Figure 4B). Similar effects were observed in intranasal administered groups to extend the center time ratio and mean speed (Figures 4C, D). In NOR experiments, the new object recognition index of APP/PS1 mice significantly decreased (*p* < 0.001) compared with the WT group. After intranasal administration of OST, lower OST/BO, and OST/BO, the index increased by 142.57% (*p* < 0.05), 179.97% (*p* < 0.001), and 172.14% (*p* < 0.01) (Figure 4E). Similarly, the percentage of spontaneous alternation behaviors (SAB) in the APP/PS1 mice group was lower than that of the WT group (*p* < 0.001). After intranasal administration of OST, lower OST/BO, and OST/BO, the percentage of SAB increased by 111.95% (*p* < 0.01), 122.68% (*p* < 0.001), and 97.92% (*p* < 0.01) (Figure 4F). During the training period of the MWM test, after intranasal treatment with OST, lower dose OST/BO, and OST/BO thermosensitive gel, the APP/PS1 mice exhibited improved memory retention by taking a shorter time to reach the hidden platform on Day 6 (Figure 5A). Likewise, the escape latency on Day 5 decreased by 25.64% (*p* < 0.05), 34.03% (*p* < 0.01), and 38.89% (*p* < 0.001) for OST, lower dose OST/BO, and OST/BO intranasal administration (Figure 5B). Compared with the APP/PS1 group, the times for crossing the platform increased by 3.9 times (*p* < 0.05), 6.5 times (*p* < 0.05), and 7.5 times (*p* < 0.05) under intranasal administration (Figure 5C). Similar results of times spent in the target quadrant were also found to be increased 134.58% (*p* < 0.05), 283.63% (*p* < 0.001), and 249.72% (*p* < 0.001) after intranasal administration with OST, lower dose OST/BO, and OST/BO, respectively (Figure 5D).

### 3.5 Anti-neuronal apoptosis effect of OST/BO thermosensitive gel via intranasal administration

**FIGURE 6:**
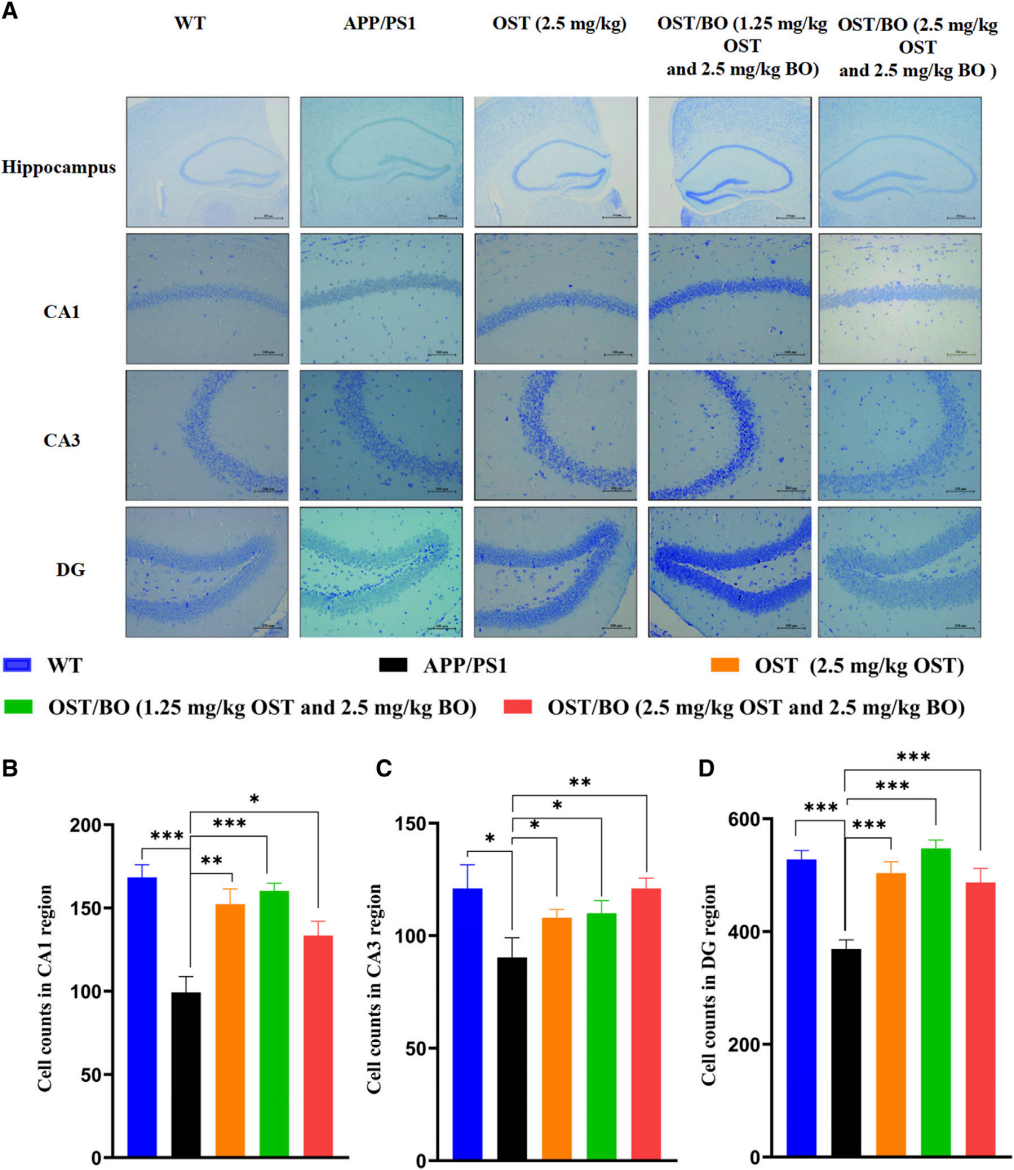
**(A)** Nissl’s staining in hippocampus, including CA1, CA3, and DG regions. **(B–D)** Statistical analysis of neuron number in CA1, CA3, and DG regions in each group. Statistical significance denoted as *p < 0.05, **p < 0.01, ***p < 0.001, (*n* = 3).

From the Nissl staining results, the number of intact neurons of the APP/PS1 group compared with the WT group reduced in the regions of CA1 (*p* < 0.001), CA3 (*p* < 0.05), and DG (*p* < 0.001) (Figure 6A). As expected, the number of intact neurons significantly increased under intranasally treated OST/BO gel. Specifically, the neurons in CA1 increased 50% (*p* < 0.01), 55% (*p* < 0.001), and 45% (*p* < 0.05) after intranasal treatment with OST (2.5 mg/kg in gel), lower OST/BO (1.25 mg/kg in gel), and OST/BO (2.5 mg/kg) (Figure 6B). The same results were observed in the regions of CA3 and DG (Figures 6C, D).

### 3.6 Aβ clearance effects of OST/BO thermosensitive gel via intranasal administration

**FIGURE 7:**
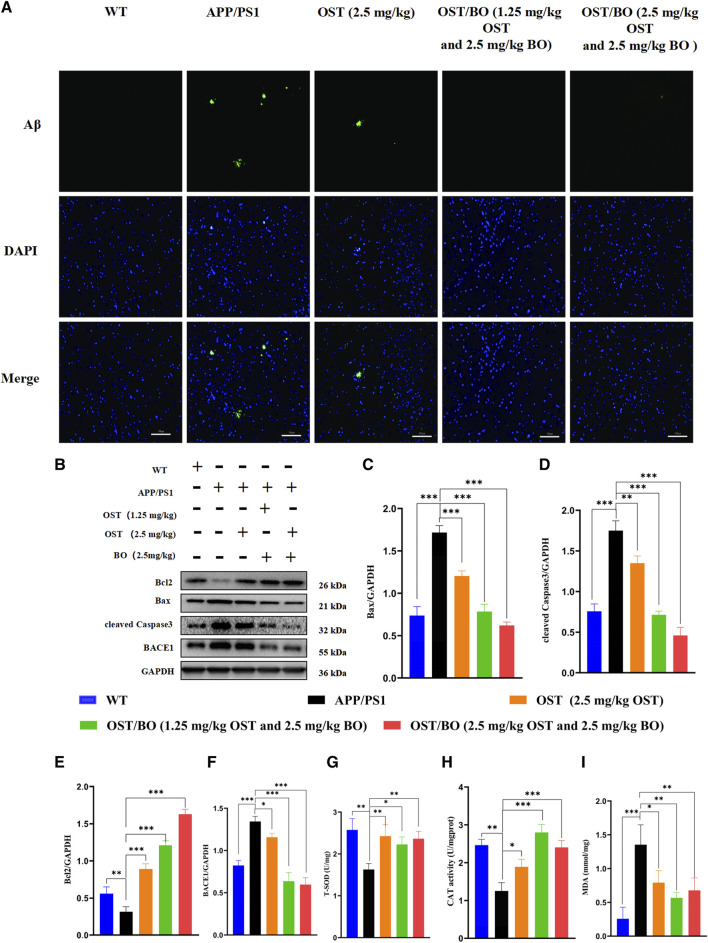
**(A)** Fluorescence images of amyloid plaques in Th-S. **(B)** WB bands of Bcl-2, Bax, BACE1, and cleaved caspase-3 in the hippocampus. **(C–F)** Statistical analysis of Bcl-2, Bax, BACE1, and cleaved caspase-3 in hippocampus. **(G–I)** T-SOD, CAT, and MDA detection results in the hippocampus of mice in each group. Data presented as means ± SD; statistical significance denoted as *p < 0.05, **p < 0.01, ***p < 0.001, (*n* = 3).

Th-S staining showed that amyloid plaques overexpressed in the brains of APP/PS1 mice compared to the WT group. According to the fluorescent results, lower dose OST/BO and OST/BO gel with intranasal administration significantly eliminated amyloid plaques, which exhibited much better efficiency than OST gel alone (Figure 7A). Afterward, the expression of typical proteins responsible for neuron loss including Bcl-2, Bax, and cleaved caspase-3 was analyzed using WB experiments. In the brains of APP/PS1 mice, Bax, and caspase-3 highly expressed while anti-apoptotic Bcl-2 decreased compared with the WT group. Under intranasal administration of OST, lower dose OST/BO, and OST/BO gel, Bcl-2 increased while Bax and cleaved caspase-3 were downregulated, and BACE1 was suppressed (Figures 7B–F). Accordingly, T-SOD activity was upregulated by 44.76% (*p* < 0.05), 36.88% (*p* < 0.05), and 45.28% (*p* < 0.01) with OST, lower OST/BO, and OST/BO intranasal treatment (Figure 7G). Likewise, CAT expression increased by 50.90% (*p* < 0.05), 123.56% (*p* < 0.001), and 92.09% (*p* < 0.01) in the hippocampus of APP/PS1 mice (Figure 7H). Furthermore, MDA decreased by 41.51% (*p* < 0.05), 58.14% (*p* < 0.05), and 50.01% (*p* < 0.05) after intranasal administration of OST and OST/BO (Figure 7I).

## 4 Discussion

The primary site of AD is in the brain, where, according to ancient TCM theory, “kidney deficiency” and “brain empty” are performed as the main pathological features. Many drugs in TCM formulas are guided to the brain under the compatibility rule of Emperor–Minister–Assistant–Courier. However, intracerebral bioavailability after oral administration is always unsatisfactory for AD treatment, which is impaired by the hepatic/intestinal first-pass effect and BBB defense. As a noninvasive method for brain delivery, intranasal administration could avoid the first-pass effect and bypass the BBB, enabling therapeutic substances direct access to CNS. The intranasal-brain route provides a larger surface area for drug absorption and also reduces untargeted administration and systemic exposure (Bhattamisra et al, 2020; Bors et al, 2020); it, therefore, exerts a faster specific therapeutic effect for brain diseases like AD (Yang et al, 2020).

As a demonstrative example here, OST has been proven to prevent glutamate-induced neurotoxicity and neuronal loss to effectively retard AD symptoms (Shao-Heng et al, 2017; Chu et al, 2020). However, poor water solubility and lower bioavailability restrain the pathological effect of OST (Ma et al, 2016). In this study, an *in situ* thermosensitive gel was prepared for intranasal administration in which OST was dissolved with a maximum solubility of 12.5 mg/mL. The gelation temperature was adjusted at 33°C in the consideration of the physiological temperature in the nasal cavity of mice (Figures 1A, B). The released behavior of OST (100%) with 5 h also guaranteed its capability for drug absorption in the nasal cavity, either using OST or OST/BO gel (Figures 1C, D).

The pharmacokinetic experiments in the oral administration groups showed no significant difference in OST concentration in rat plasma or CSF, either treated with OST or OST/BO (in corn oil). This indicates that BO could not improve the uptake of OST in blood circulation or into the brain as we proposed, probably owing to the relatively lower dose of BO (2.5 mg/kg) in this study, which failed to conquer the first-pass effect and penetrate BBB. A former study found that oral BO could improve the plasma bioavailability of OST, but the dosage of BO reached 400 mg/kg (Luo et al, 2017). This may be caused by a higher dose of BO inhibiting the action of the P-gp transporter in intestinal mucosa and CYP450 metabolism in the liver. In contrast, after intranasal administration of OST and OST/BO (2.5 mg/kg, in gel), the AUC_0-t_ and C_max_ of OST in plasma were much higher than oral administration (Table 1), which demonstrated that thermosensitive gel-based intranasal absorption could avoid the first-pass effect as well as intestinal barrier function which facilitated OST into the blood. It is interesting that the concentration of OST in plasma was almost identical between OST and OST/BO intranasal administration groups, indicating that BO had not yet achieved OST penetration into the blood in the nasal cavity (Figure 2A; Table 1). However, regarding OST concentration in CSF, after OST/BO intranasal administration the AUC_0-t_ and C_max_ increased significantly over OST alone (Figure 2B; Table 2). The same trend also appeared in lower OST (1.25 mg/kg) and OST/BO (1.25 mg/kg for each) by intranasal administration (Supplementary Figure S1), but the difference was not as significant as 2.5 mg/kg. This indicates that BO as a “courier” increased the intracerebral bioavailability of OST, probably because BO evacuated the intranasal-brain route (Lochhead and Thorne, 2012). Such a hypothesis was soon evidenced by HE staining of the nasal mucosa in SD rats and APP/PS1 mice (Supplementary Figure S2). Compared with OST gel, the intercellular space within the nasal mucosa cells was widely divided under OST/BO gel intranasal administration (Figure 3A). The speculated mechanism was that the intranasal administration of BO suppressed the tight junction protein expression, such as ZO-1 and occludin (Figures 3B–D) to thus open the gap between nasal epithelial cells and expand the nose-to-brain route. Such findings agree with Chen et al. (2013) and Chen et al. (2014)that BO could interpret nasal administration to improve the entry of other drugs into the brain by loosening the intercellular tight junction of MDCK, MDCK-MDR1, and nasal epithelial cells.

The pharmacological role of OST guided by BO toward AD was furtherly verified by intranasal administration. The behavioral test findings of APP/PS1 transgenic mice showed that the administered groups of OST/BO gel exhibited much better effects than the single OST group (Figures 4, 5), but there was no significant difference between the 1.25 mg/kg and 2.5 mg/kg of OST/BO groups. Intranasally administered OST and OST/BO suppressed cleaved caspase-3 expression and increased the ratio of Bcl-2/Bax, improved expression of T-SOD and CAT, reduced MDA, inhibited neuron apoptosis, and decreased Aβ level by inhibiting BACE1 expression (Figures 6, 7). Such results further proved the function of BO as a “courier” guiding OST delivery to the brain and benefitting AD treatment, based on the TCM Emperor–Minister–Assistant–Courier theory. However, there was no significant difference in the anti-AD effect of 1.25 mg/kg and 2.5 mg/kg OST/BO gel. More doses and many other bioactive constituents should be investigated to explain the modern connation of TCM compatibility, and such work is underway.

In order to improve the intracerebral bioavailability of drugs for AD treatment, an *in situ* thermosensitive gel mixed with OST and BO was prepared in this work. By inhibiting ZO-1 and occludin expression, BO opened up the intercellular space and loosened the tight junctions of the nasal mucosa, facilitating nose-to-brain delivery and directing OST to the brain target. The anti-AD effects of OST/BO thus exhibited much better efficiency in improving cognitive impairment in APP/PS1 mice than OST alone. This study not only provided an alternative drug-delivery route with higher intracerebral bioavailability for AD treatment but also showed the scientific compatibility of Chinese herbal compounds.

## Data Availability

The original contributions presented in the study are included in the article/Supplementary Material; further inquiries can be directed to the corresponding authors.
