# Supplementary material for: Osthole/borneol thermosensitive gel via intranasal administration enhances intracerebral bioavailability to improve cognitive impairment in APP/PS1 transgenic mice
Source: Front Pharmacol. 2023 Jul 13;14:1224856. doi: 10.3389/fphar.2023.1224856 (PMC10373789; doi:10.3389/fphar.2023.1224856)
Supplement: Supplementary file 1 [file DataSheet1.docx]

Supplementary Material

Osthole/Borneol thermosensitive gel via intranasal administration enhanced intracerebral bioavailability to improve cognitive impairment in APP/PS1 transgenic mice

Fanchang Wu, Mingjun Huang, Xue Zuo, Ruiye Xie, Jinman Liu, Junyu Ke, Weirong Li, Qi Wang* and Yong Liang*

***Correspondence:** Yong Liang: liangyong@gzucm.edu.cn; Qi Wang: wangqi@gzucm.edu.cn

# Supplementary Figures and Tables

## Supplementary Figures

**
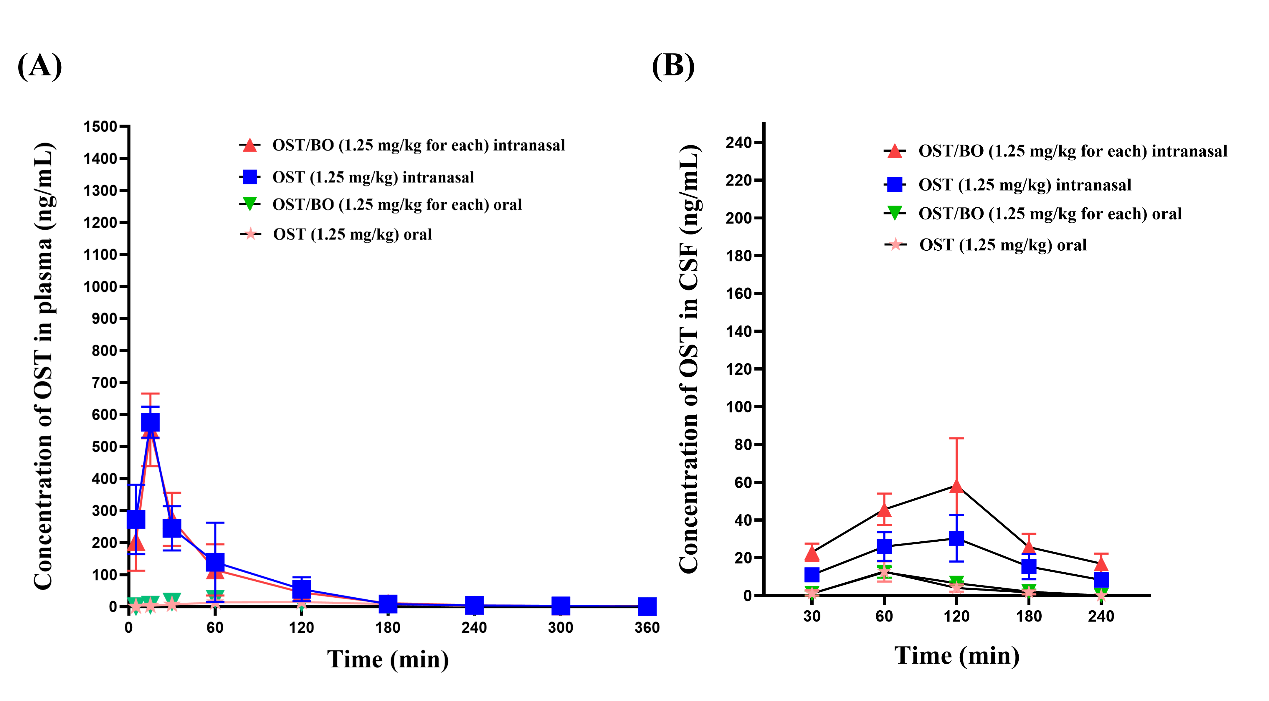
**

**Supplementary Figure 1.** The curve of average concentration for OST (ng/mL) vs sampling time (min) in rat plasma and CSF determined by LC-MS/MS. (A) The content of OST in rat plasma after oral and intranasal administration (n = 6). (B) The content of OST in rat CSF after oral and intranasal administration (n = 6).


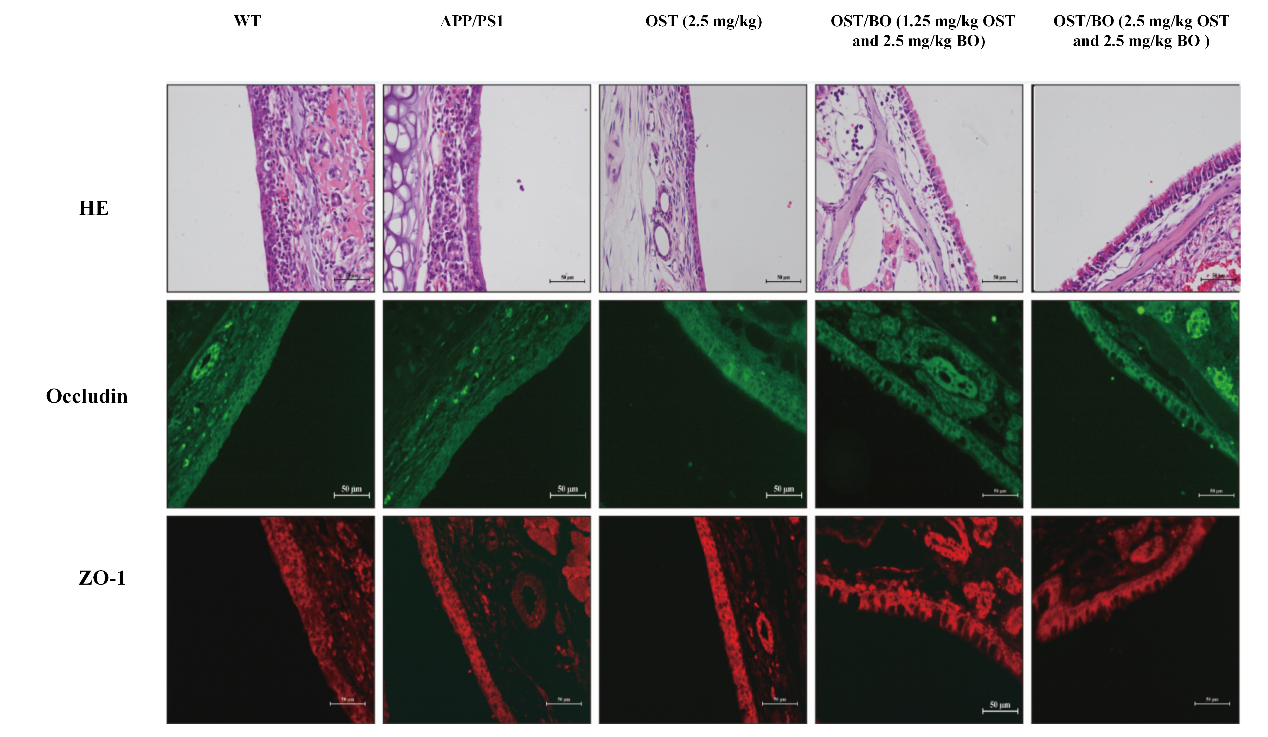


# Supplementary Figure 2. Pathological images of nasal mucosa stained with HE and immunohistochemistry images of ZO-1 and Occludin in the nasal mucosa of APP/PS1 mice.

## Supplementary Table

| **Table Supplementary 1**  Various proportions of P407, P188 and PEG300 were optimized to obtain gelation temperature with Box-Behnken design (n = 3). | | | | | |
| --- | --- | --- | --- | --- | --- |
| P407 (%) | P188 (%) | | PEG 300 (%) | | Gelation temperature (°C) |
| 24 | | 10 | | 12.5 | 16 |
| 20 | | 7 | | 6 | 32 |
| 22 | | 7 | | 6 | 30 |
| 24 | | 7 | | 12.5 | 27 |
| 24 | | 7 | | 6 | 23 |
| 20 | | 3 | | 12.5 | 26 |
| 24 | | 6.6 | | 0 | 27 |
| 20 | | 10 | | 6 | 35 |
| 24 | | 3 | | 6 | 18 |
| 20 | | 7 | | 0 | 38 |
| 24 | | 10 | | 0 | 28 |
| 22 | | 3 | | 6 | 22 |
| 24 | | 10 | | 6 | 21 |
| 22 | | 3 | | 0 | 27 |
| 20 | | 7 | | 12.5 | 38 |
| 20 | | 3 | | 6 | 27 |
| 20 | | 10 | | 12.5 | 37 |
